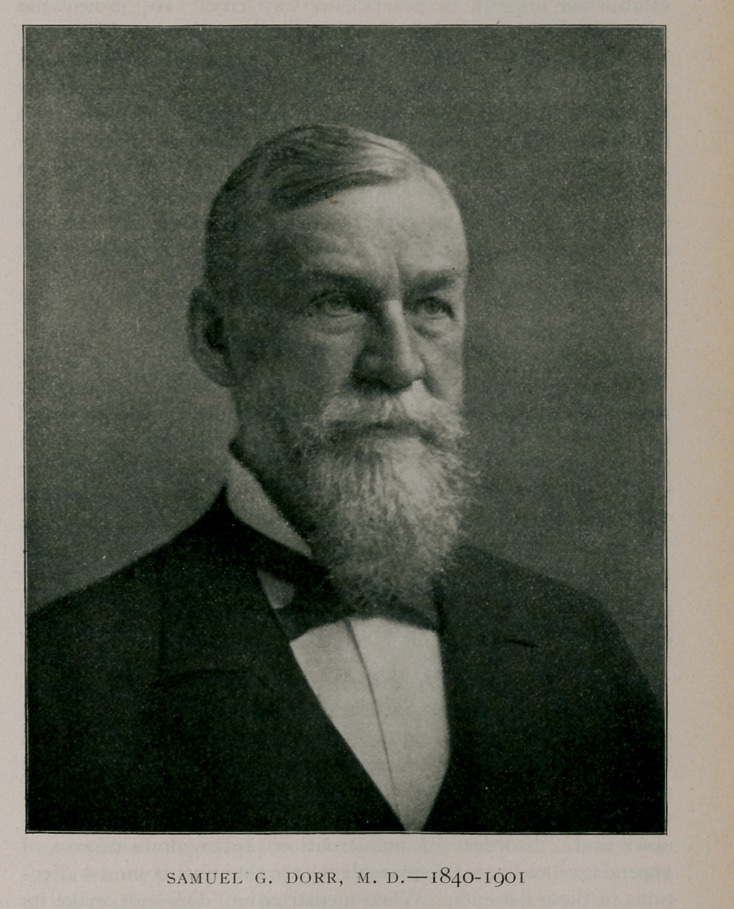# A Century of Medical History in the County of Erie.—1800–19001Note.—The last regular chapter of this history was published in the Buffalo Medical Journal, April, 1899, which brought the record down to that time. Supplementary chapters are being presented from time to time that the history may be carried from that date forward to the end of the XIXth. Century, thus completing it.—W. W. P.

**Published:** 1902-11

**Authors:** William Warren Potter

**Affiliations:** Buffalo, N.Y.


### A Century of Medical History in the County of Erie.—1800-1900.[1]

By WILLIAM WARREN POTTER, M. D., Buffalo, N. Y.

*Pioneer Physicians—Medical Societies—Medical Colleges—Hospitals—Medical Journals—Women Physicians—History of Homeopathy—Medical Officers of the Civil War—Individual Members of the Profession.*

### SUPPLEMENT.

### X.—Medical Society of the County of Erie.

AT THE 78th annual meeting, held January 10, 1899, the following-named physicians were admitted to membership: John Hudson Grant, J. C. Clemesha, Emil Lustig, Abra-

1. Note.—The last regular chapter of this history was published in the Buffalo Medical Journal, April, 1899, which brought the record down to that time. Supplementary chapters are being presented from time to time that the history may be carried from that date forward to the end of the XIXth. Century, thus completing it.—W. W. P.

ham L. Weil, Mary Clayton, James W. Charters, N. L. Burnham, Christ A. Weinbach and Charles R. Borzilleri.

The officers chosen were: president, J. B. Coakley; vice-president, E. H. Ballou, of Gardenville; secretary, F. C. Gram; treasurer, Edward Clark; librarian, W. C. Callanan; censors,

JOSEPH C. GREENE, M. D.—1829-1899.

From THE ILLUSTRATED BUFFALO EXPRESS.
*Copyright*, 1899, by Geo. E. Matthews & Co.

J. B. Coakley, Irving W. Potter, Thomas F. Dwyer, Charles E. Congdon, F. E. Fronczak.

At the semiannual meeting June 13, the following-named were admitted: H. B. Brownell, Edward J. Kiepe, William B. May, Nelson W. Wilson, Montressor Axford, H. R. Gaylord, Charles A. Brownell and Charles F. Durand.

The deaths during the year were: Dr. A. H. Crawford, January 20, aged 58 years; Joseph C. Greene, January 3, aged 69 years; Dr. L. P. L. Parker, Akron, January 1, aged 70 years; Dr. Wesley C. Earl, June 19, aged 64 years; Dr. Edward E. Stanbro, November 5, aged 30 years; Dr. W. E. Robbins, Hamburg, December 5, aged 39 years; Andrew J. Volker, December 11, aged 28 years.

Joseph C. Greene, was born at Lincoln, Vt., July 31, 1829, where he resided for sixteen years. His preliminary education was received in public school and academy.

He received the degree of M. D. from Albany Medical College in 1855, after which he practised for a short time in Charlotte and in 1863 came to Buffalo.

He joined the society in 1864 and was its president in 1884. He was a member and at one time president of the Buffalo Historical Society. During the years 1873 and 1874 he was district physician of the Buffalo Board of Health, and in 1885 he served as alderman from the then second ward.

In company with his brother, Dr. S. S. Greene he made a tour of the world, leaving Buffalo, September 3, 1888, spending fourteen months in travel through the eastern countries and Europe. A valuable collection of relics, curios and antiquities, made in Egypt, Syria and Assyria, was, on his return, given to the Buffalo Historical Society.

Wesley Clark Earl was a native of Vermont, where he passed a portion of his early life. He began the study of medicine with Dr. M. S. Kittinger, of Lockport, entered the University of Buffalo for a year and graduated from Bellevue Hospital Medical College in 1864. Soon after graduation he was appointed acting assistant surgeon in the United States Army during the civil war and was first assigned to duty at Fort Schuyler. At a later period his assignment was changed to Elmira, where he served in caring for confederate prisoners under Dr. William C. Wey, who was surgeon in charge.

After the war Dr. Earl located at Pekin, Niagara County, where he practised medicine nine years. He removed to Buffalo in 1874, where he continued in practice until his death. He joined the society in 1875. He was prominent as a physician, representing the best type of family doctor. He served as an officer of the Riverside Methodist Episcopal Church, and was a member of several lodge societies, and of the Society of Vermonters.

W. E. Robbins, of Hamburg, was born in Iowa, on November 7, 1860; he came with his parents to Erie County when a small boy, the family settling in North Evans. He lived there until he entered the University of Buffalo, graduating with the class of '85. His standing in the profession was high and he acquired a large practice, especially in the southern part of the county. He was a member of the staff of the Erie County

W. E. ROBBINS, M. D.—1860-1899.

From the ILLUSTRATED BUFFALO EXPRESS.
*Copyright*, 1900, by Geo. E. Matthews & Co.

Hospital, and of various medical societies. He had been health officer of the town of Hamburg and held a like position in the village at the time of his death. He joined the society in 1887.

Dr. Robbins was identified with every public movement in his village and had the highest esteem of all its residents.

At the 79th annual meeting, held January 9, 1900, physicians were admitted to membership as follows: Felix Hintz, Catherine E. Kelly, Daniel P. Doyle, William J. Deane, James W. Nash and Ernest W. Ewell.

Officers were elected as follows: president, E. H. Ballou, of Gardenville; vice-president, Wm. C. Phelps; secretary, Franklin C. Gram; treasurer, Edward Clark; librarian, W. C. Callanan; censors, J. B. Coakley, chairman, H. R. Hopkins, I. W. Potter, T. F. Dwyer and F. E. Fronczak; committee on membership, William Warren Potter, chairman, G. W. McPherson and J. J. Walsh.

At the semi-annual meeting June 12, the following-named were admitted: Chauncey P. Smith, William R. Little, Robert K. Grove and Francis M. O'Gorman.

During the year the following deaths occurred: Elias T. Dorland, February 20, aged 67 years; A. R. Wright, February 24, aged 74 years; George S. Palmer, April 16, aged 38 years; L. P. Dayton, May 14, aged 80 years; George W. Lewis, July 24, aged 38 years.

Elias T. Dorland was born at Oswego, April 12, 1832, and his father Joseph Dorland, being a prominent physician in that region of the state. His ancestry were of the old Dutch stock, so famous in the Mohawk Valley, but in early life he came to Erie County, where his preliminary education was received in the public schools and at Springville Academy. He began his medical studies in Buffalo and attended the Medical Department of the University for a year. Afterward he continued his studies in and was graduated from the University of Michigan. After his graduation he was appointed resident physician at the Erie County Almshouse, which post he held for two years. After the expiration of his term of service he engaged in private practice at La Grangeville, Dutchess County, where he remained for twelve years. In 1866 he returned to Buffalo where he resided until his death. Dr. Dorland was one of the best known and most respected of the older physicians in Buffalo.

He joined the society in 1869, and served as its president in 1886; he was also a member of the Medical Union, of which also he had served as president; and a member of the New York State Medical Association. During the later years, owing to failing health, he did not take an active part in medical affairs, yet he always maintained a deep interest in the guild.

In 1888, Dr. Dorland became a candidate for the Assembly and his successful opponent was William F. Sheehan, who

ELIAS T. DORLAND, M. D.

became speaker, and afterward lieutenant governor. He was a member of the American Legion of Honor, and a member of the Delaware Avenue Baptist Church.

Lewis P. Dayton was born in Eden, Erie County, in 1819, was graduated from Springville Academy in 1840 and from the Medical College at Geneva in 1845, in which year he settled in Buffalo. From that time on he ranked as one of Buffalo's leading physicians. He was identified with many movements begun by the Medical Society of the County of Erie, and was

LEWIS P. DAYTON, M. D.—1819-1899

From the ILLUSTRATED BUFFALO EXPRESS.
*Copyright,* 1899, by Geo. E. Matthews & Co.

recognised as the dean of the profession of medicine in Buffalo during his later years.

In 1845, Dr. Dayton was elected school commissioner for the town of Black Rock. In 1849 he joined the Medical Society of the County of Erie, was elected vice-president in 1858, and served as president in 1859. During the years 1855, 1856, 1857, 1858, 1862, 1863 and 1864, he represented the old

12th Ward (now the 25th) in the Board of Aldermen. He was Member of Assembly from the Third District in 1865 and 1866, and was Mayor in 1874. He was twice elected County Clerk and served one term as County Treasurer.

FRANK WAYLAND ABBOTT, M. D.—1842-1901.

At the 80th annual meeting, held January 8, 1901, the following-named physicians were admitted to membership: Henry C. Lapp, of Clarence; George N. Jack, of Depew; Wm. C. Jolls, of Orchard Park; Frederick H. Ehinger, of Ebenezer; Prescott

LeBreton, Grosvenor R. Trowbridge and James J. Mooney, of Buffalo.

The election of officers resulted as follows: president, Wm. C. Phelps, Buffalo; vice-president, Walden M. Ward, North Collins; secretary, Franklin C. Gram; treasurer, Edward Clark; librarian, Wm. C. Callanan; board of censors, J. B. Coakley, chairman, and Henry R. Hopkins, Irving W. Potter, F. E. Fronczak, and Henry Lapp, of Clarence.

At the semi-annual meeting held June 11, the following-named physicians were admitted to membership: J. Henry Dowd, Samuel H. Lynde, George H. McMichael and Theodore V. Bauer.

The deaths for the year were: William H. Pleuthner, January 17, aged 32 years; Henry Nichell, February 14, aged 79 years; J. Stone Armstrong, March 3, aged 61 years; Frank W. Abbott, April 9, aged 59 years; Samuel G. Dorr, April 28, aged 61 years; Edward L. Groess, June 19, aged 38 years; Frank P. Bingham, August 2, aged 28 years; Edward P. Hay, August 10, aged — years; Jessie Shepard, August —, aged — years.

Frank Wayland Abbott for years occupied a prominent place in the profession of medicine, distinguished for his skill, judgment, accuracy and integrity. He was one of the most prominent ophthalmologists and otologists in Buffalo, serving in that capacity on the staff of the General Hospital, at the Erie County Charity Eye, Ear and Throat Hospital, and as a member of the Board of Pension Surgeons. For many years he was chairman of the executive committee of the Buffalo General Hospital Training School for Nurses.

Dr. Abbott was also a lay reader of the Episcopal Church. He graduated in medicine at the University of Buffalo in 1866 and joined the society the same year.

Samuel Griswold Dorr came of ancient German lineage and Edmund Dorr, one of his ancestors, came from England to Connecticut early in the XVIIth century. Captain Matthew Dorr, another of his ancestors, distinguished himself in the war of the revolution. Edmund Dorr, before alluded to, married a member of the distinguished Griswold family, from which Dr. Dorr derived his middle name.

Dr. Dorr was born at Dansville, N. Y., May 30, 1840, where he spent a portion of his early life. He received his preliminary education at Nunda Academy in this state and at the Albion State Academy in Wisconsin. Upon the outbreak of the civil war he enlisted in the union army, but almost immediately he was seized with diphtheria, which invalided him for a year, hence he was unable to go to the field. In 1863, however, he was appointed a recruiting agent in Livingston County, where

SAMUEL G. DORR, M. D.—1840-1901

he rendered valuable service during the remainder of the war period. After peace came he engaged in the oil refining business in Pennsylvania and established a barrel factory as a collateral branch of the business. These occupations, however, were not suited to his desires and he turned his attention to medicine, following in this way the traditions of his family. In 1873 he came to Buffalo and entered the University as a student. He took his doctorate degree with honor in 1875 and immediately established himself in practice in this city.' He joined the society in 1876.

He served two terms as police surgeon, was at one time a member of the consulting staff of the Sisters of Charity Hospital and a member of the several local medical societies. In 1888 he served in the national republican convention at Chicago. In 1899 he was appointed postmaster of Buffalo by President McKinley and entered upon the duties of that office April 1, in that year. He faithfully served in that capacity until his sudden death from angina pectoris terminated his official career.

## Figures and Tables

**Figure f1:**
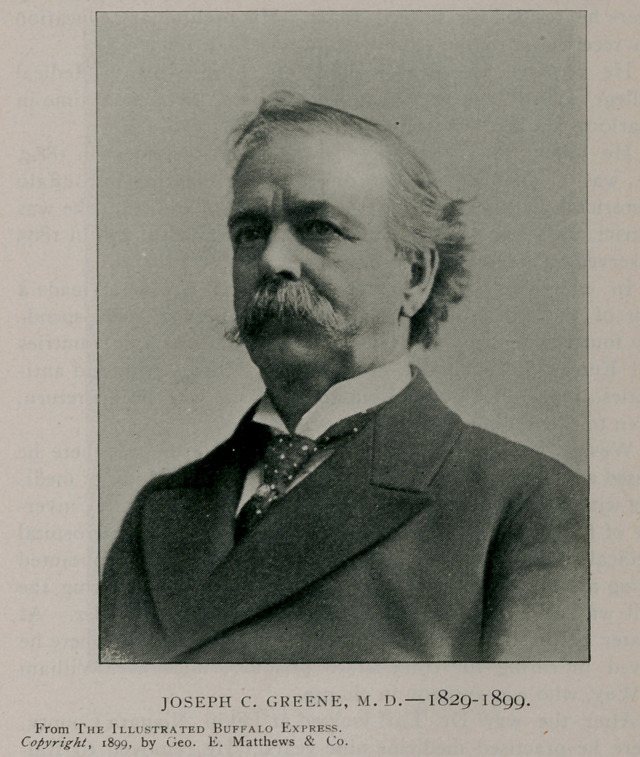


**Figure f2:**
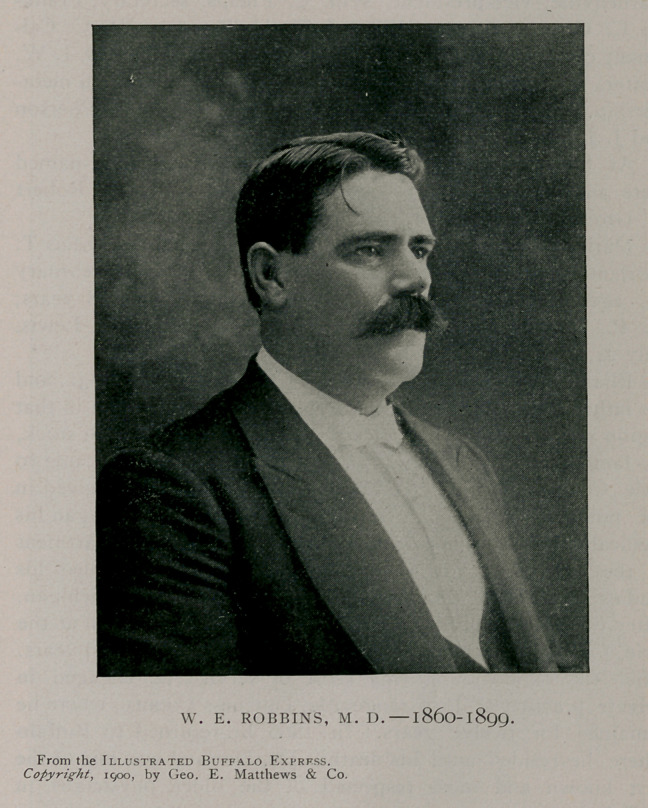


**Figure f3:**
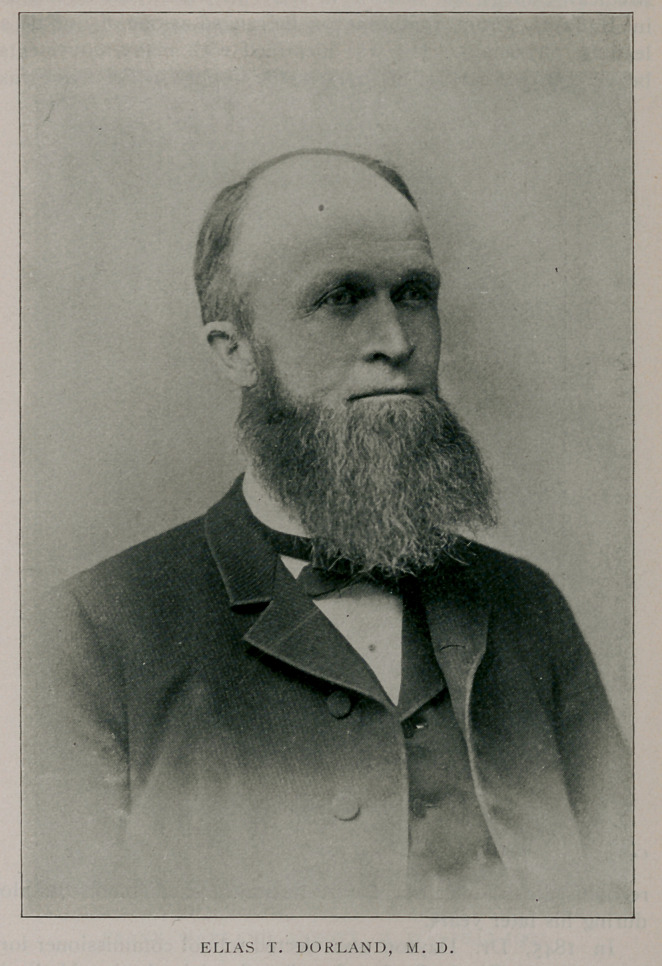


**Figure f4:**
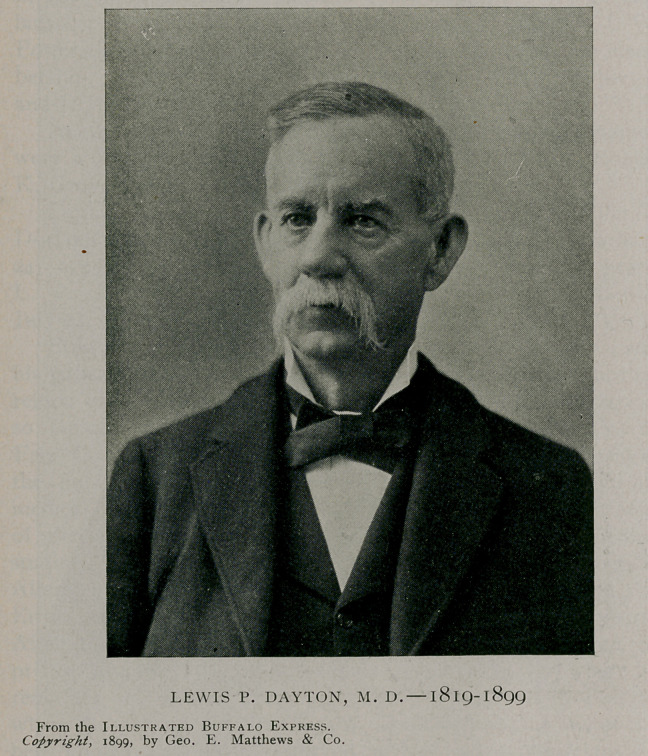


**Figure f5:**
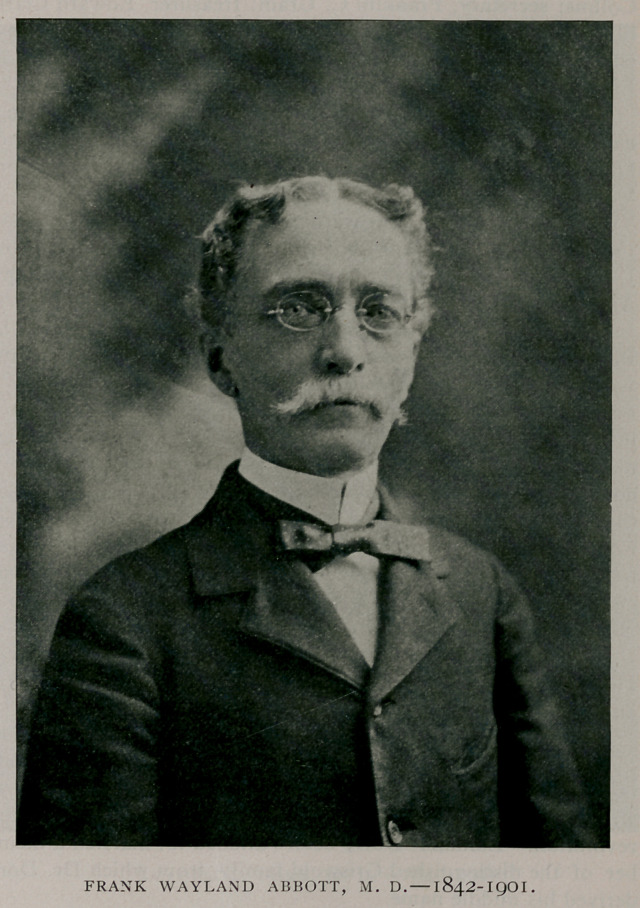


**Figure f6:**